# Modular nature of simian foamy virus genomes and their evolutionary history

**DOI:** 10.1093/ve/vez032

**Published:** 2019-10-16

**Authors:** Pakorn Aiewsakun, Léa Richard, Antoine Gessain, Augustin Mouinga-Ondémé, Philippe Vicente Afonso, Aris Katzourakis

**Affiliations:** 1 Department of Microbiology, Faculty of Science, Mahidol University, Bangkok 10400, Thailand; 2 Unité d’Epidémiologie et Physiopathologie des Virus Oncogènes, Institut Pasteur, UMR3569 CNRS, Paris, France; 3 Université Paris Diderot – Paris7, Sorbonne Paris Cité, Paris, France; 4 Unité des Infections Rétrovirales et Pathologies Associées, Centre International de Recherches Médicales de Franceville (CIRMF), Franceville, Gabon; 5 Department of Zoology, University of Oxford, South Parks Road, Oxford OX1 3SY, UK

**Keywords:** foamy virus, simian foamy virus, mandrill, mandrill foamy virus, co-evolution, co-speciation, modular genomic evolution, time-dependent rate phenomenon

## Abstract

Among all known retroviruses, foamy viruses (FVs) have the most stable virus–host co-speciation history, co-diverging in concert with their vertebrate hosts for hundreds of millions of years. However, detailed molecular analyses indicate that different parts of their genome might have different evolutionary histories. While their polymerase gene displays a robust and straightforward virus–host co-speciation pattern, the evolutionary history of their envelope (*env*) gene, is much more complicated. Here, we report eleven new FV *env* sequences in two mandrill populations in Central Africa, geographically separated by the Ogooué River into the North and the South populations. Phylogenetic reconstruction of the polymerase gene shows that the two virus populations are distinct, and each contains two variants of *env* genes co-existing with one another. The distinction between the two *env* variants can be mapped to the surface domain, flanked by two recombination hotspots, as previously reported for chimpanzee and gorilla FVs. Our analyses suggest that the two *env* variants originated during the diversification of Old World monkeys and apes, ∼30 million years ago. We also show that this *env* gene region forms two phylogenetically distinct clades, each displaying a host co-divergence and geographical separation pattern, while the rest of the genome of the two strains is phylogenetically indistinguishable in each of the host-specific groups. We propose possible evolutionary mechanisms to explain the modular nature of the FV genome.

## 1. Introduction

Foamy virus (FV) is a unique subgroup of retroviruses that forms a single *Spumaretrovirinae* subfamily basal to all known contemporary retroviruses (Hayward, Cornwallis, and Jern 2015). It comprises five genera, namely *Simiispumavirus* genus (a group of simian FVs, SFVs), *Prosimiispumavirus* genus (prosimian FVs), *Bovispumavirus* genus (bovine FVs), *Equispumavirus* genus (equine FVs), and *Felispumavirus* genus (feline FVs) (Khan et al. 2018). Epidemiological surveys have revealed that the host range of FVs covers a wide range of mammals, from non-human primates to cows and horses. The discoveries of FV-like endogenous retroviruses later suggested that they are, or at least were, capable of infecting a wide range of vertebrates, including fish and amphibians (Llorens et al. 2009; Han and Worobey 2012; Schartl et al. 2013; Ruboyianes and Worobey 2016; Aiewsakun and Katzourakis 2017). Furthermore, long-term evolutionary studies of FVs, together with their endogenous counterparts, have shown that they have a remarkably stable co-speciation history with their hosts. This extraordinary evolutionary feature allowed researchers to trace back their origin to the diversification of vertebrates, almost half a billion years ago (Aiewsakun and Katzourakis 2017). This unique feature also enabled researchers to model how viruses evolve through time over a timescale of millions of years (Aiewsakun and Katzourakis 2015), contributing to the shift in our understanding of virus evolution.

Although FVs may appear to have an extremely stable co-speciation history with their hosts over long timescales, epidemiological surveys at the sub-species level suggested that their interaction might not be simple. Coinfections with multiple FV strains, or even FV species, can occur. For example, it has been recorded that some chimpanzees were coinfected with multiple Old World monkey and ape (OWMA) SFVs (Leendertz et al. 2008; Liu et al. 2008), and this can lead to recombination between genetically divergent strains (Liu et al. 2008; Feeroz et al. 2013; Galvin et al. 2013). Moreover, molecular studies have shown that their *gag* and accessory genes can be polymorphic, varying among FVs originating from different host individuals, or even among particles present within the same host (Rua et al. 2012). Polymorphisms in *env* genes have also been recorded in SFVs that infect chimpanzees (simian foamy virus Pan troglodytes schweinfurthii, SFVpsc; simian foamy virus Pan troglodytes verus, SFVpve; and simian foamy virus Pan troglodytes troglodytes, SFVptr), gorillas (simian foamy virus Gorilla gorilla gorilla, SFVggo), macaques (simian foamy virus Macaca cyclopis, SFVmcy; simian foamy virus Macaca mulatta, SFVmmu), and Cercopithecini monkeys (simian foamy virus Chlorocebus aethiops, SFVcae; simian foamy virus Cercopithecus nictitans, SFVcni), segregating into two variants, and cocirculating within the same host populations (Galvin et al. 2013; Richard et al. 2015).

This study reports an epidemiological survey and molecular analyses of SFVs circulating among mandrills (simian foamy virus Mandrillus sphinx, SFVmsp) in Gabon and Cameroon. As previously determined for other SFVs (Galvin et al. 2013; Richard et al. 2015), phylogenetic analyses showed that the *env* gene of SFVmsp displays two variants that cocirculate within the same host population. Most of the genetic diversity could be mapped to the surface domain (SU) region flanked by two recombination hotspots (RHs). In contrast, phylogenetic analyses of the *pol* gene and the conserved regions of the *env* gene showed segregation of SFVs by hosts, consistent with the well-known notion of FV–host co-speciation. By taking the recently established time-dependent rate phenomenon (TDRP) into account (Aiewsakun and Katzourakis 2015, 2016), we estimated the origin of the two *env* variants to be ancient, dated back ∼30 million years (Myr) ago (Mya). We propose evolutionary scenarios that can explain these findings.

## 2. Results

### 2.1 Epidemiological survey of foamy virus among mandrills in Gabon and Cameroon

We previously identified roughly a hundred mandrills infected with a specific SFV strain (Calattini et al. 2004; Mouinga-Ondeme et al. 2010, 2012). DNA samples of 11 of these animals were still available: 10 from Gabon and 1 from Cameroon (Table 1). DNA quality was attested by a β-globin PCR, and SFV status was confirmed by the SFV *pol-integrase* PCR, as previously described in Calattini et al. (2004) and Mouinga-Ondeme et al. (2010, 2012). All samples were found positive for SFV *pol*, including the MSP115 sample (GenBank accession number: MK014762), which had been previously found as serologically positive, but negative with the PCR assay (Mouinga-Ondeme et al. 2010). Complete SFV *env* sequences were obtained from four of these samples (two from South Gabon animals, and two from North Gabon) by the nested PCR technique (see primers used in Supplementary Table S1). In addition, the central *env* regions, corresponding to the SU, were obtained from the remaining mandrills. Sequences are available in GenBank (Table 1).

**Table 1.** FVs detected in Gabon and Cameroon mandrills.

Country	Region	Host name	Origin	Reference	GenBank accession number *pol*	Obtained *env* fragment (nt)	GenBank accession number *env*	Virus variant
Gabon	North	MSP115	Wild-born/ caught	Mouinga-Ondeme et al. (2010)	MK014762	Partial (1,346)	MK014755	SFVmsp I
Gabon	North	Md12D3F	CDP	Mouinga-Ondeme et al. (2010)	GU169767	Complete (2,949)	MK014758	SFVmsp II
Gabon	North	Md12P1	CDP	Mouinga-Ondeme et al. (2010)	GU169769	Complete (2,973)	MK014761	SFVmsp I
Gabon	North	Md13	CDP	Calattini et al. (2004)	AY583781	Partial (1,322)	MK014751	SFVmsp II
Gabon	North	Md14	CDP	Calattini et al. (2004)	AY583780	Partial (1,322)	MK014750	SFVmsp II
Gabon	North	Md15	CDP	Calattini et al. (2004)	AY583779	Partial (1,322)	MK014752	SFVmsp II
Gabon	North	Md2e	CDP	Calattini et al. (2004)	AY583776	Partial (1,322)	MK014753	SFVmsp II
Gabon	South	MSP100	Wild	Mouinga-Ondeme et al. (2012)	HQ450622	Complete (2,949)	MK014759	SFVmsp II
Gabon	South	Idiata	Wild	Mouinga-Ondeme et al. (2010)	GU169796	Partial (1,322)	MK014754	SFVmsp II
Gabon	South	MSP38	Wild	Mouinga-Ondeme et al. (2010)	GU169801	Complete (2,973)	MK014760	SFVmsp I
Cameroon		Md301L	Wild-born/ caught	Calattini et al. (2004)	AY583775	Partial (1,322)	MK014749	SFVmsp II

CDP, Centre de Primatologie; SFVmsp, simian foamy virus Mandrillus sphinx.

### 2.2 Recombination within the *env* alignment

**Figure 1.**
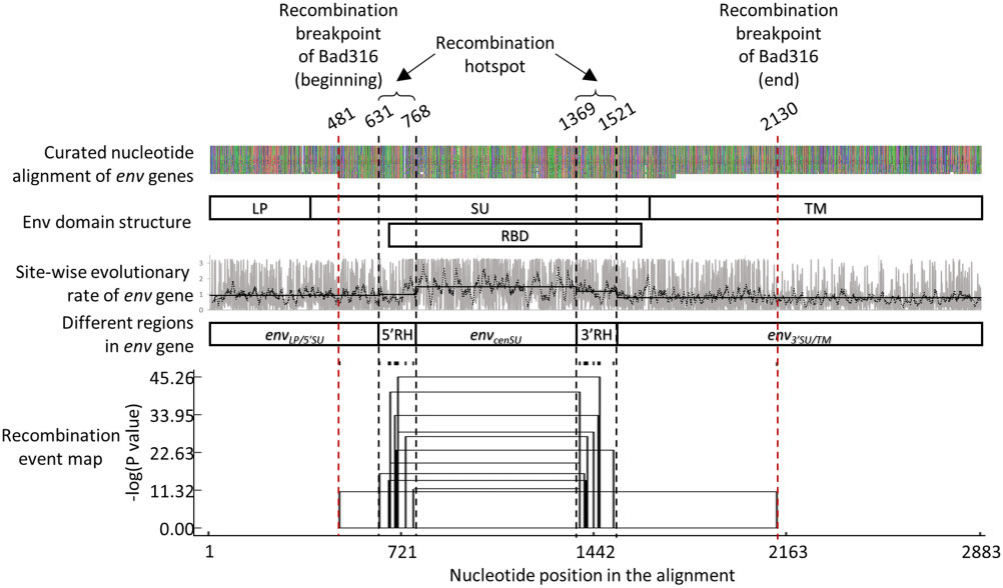
Recombination analysis. From top to bottom: 
Curated *env* alignment. The alignment contains fifty-four sequences, forty-seven of which are full-length sequences. The total length of the alignment is 2,883 nucleotides, including gaps.Schematic outline of the Env domain structure. LP, leader peptide; SU, surface domain; TM, transmembrane domain; RBD, receptor binding domain.Site-wise evolutionary rates of *env* gene. The rates are scaled such that the average rate across all sites is 1 (no units). Site-wise scaled rate values are plotted in grey. Black dotted line shows moving average, computed over a window of 10 rate estimates. Black solid lines show average values of the rate estimates within various regions of the *env* gene. *env_LP/5_*_′__*SU*_: 0.941; 5′ RH: 0.995; *env_cenSU_*: 1.491; 3′ RH: 1.209; *env_3_*_′__*SU/TM*_: 0.788.Schematic outline of the *env_LP/5_*_′__*SU*_, *env_cenSU_*, *env_3_*_′__*SU/TM*_, and RH regions of the *env* gene.Recombination event map. The plot shows estimated positions of recombination breakpoints, and the P values associated with the detected recombination signals. The tick marks above the plot indicate individual recombination breakpoints inferred. Two RHs were found; the first region is between 631 and 768 nt (5′ RH), and the second one is between 1,369 and 1,521 nt (3′ RH) of the alignment. The regions are indicated by black vertical dotted lines. We noted that, while our analyses inferred the *env* sequence ‘Bad316’ to be a recombinant, its recombination break points lie outside the detected RHs, estimated to be at 481 nt, and 2,130 nt, indicated by vertical red dotted line.

Alignments of *pol* and *env* genes were prepared for phylogenetic analyses, and potential recombination was assessed using seven programmes, including RDP, GENECONV, Chimaera, MaxChi, BootScan, SiScan, and 3Seq, implemented in the Recombination Detection Program 4 package (Martin et al. 2015). Two RHs were detected by all seven programmes in the *env* alignment at a P value <0.05, both of which were located within the Env SU-coding region (Fig. 1). From the 5′ end, the first and second hotspots lie approximately between 631 and 768 nt, and between 1,369 and 1,521 nt of the alignment, respectively. These RHs can be mapped to 7,775–7,909 nt and 8,564–8,716 nt of the prototype foamy virus (PFV; GenBank accession number Y07725) genome in their respective order, dividing the *env* genes into three parts: 1, the sequence region corresponding to the leader peptide (LP) and the 5′ portion of the SU (*env_LP/5_*_′__*SU*_), 2, the central SU coding region, specifically the receptor binding domain (RBD) (*env_cenSU_*), and 3, the coding region for the 3′ end of the SU and the transmembrane domain (TM) (*env_3_*_′__*SU/TM*_). All recombination breakpoints were inferred to lie within these two RHs, except those of Bad316, a FV of chimpanzee origin (Richard et al. 2015). Its breakpoints were estimated to be at 481 nt, and 2,130 nt of the alignment, spanning the SU and TM of the *env* gene (Fig. 1), detected by all seven programmes at a P value <0.05. In contrast, although recombination in SFVs has been observed in the *pol* gene (Liu et al. 2008), our analyses could not detect recombination within the *pol* alignment, but this could be due to the short alignment, spanning only the integrase coding region.

Site-wise evolutionary rates of the *env* gene were computed to investigate rate variation among these different regions of *env* (Fig. 1). The rates were scaled so that the average value across all sites is 1 (no units). We found that the average rate of the *env_LP/5_*_′__*SU*_ portion is 0.941 (95% highest posterior density (HPD) = 0.054–3.123), and that of the *env_3_*_′__*SU/TM*_ region is 0.788 (95% HPD = 0.054–3.063). The *env_cenSU_* region, however, seems to be much less conserved, having an average rate of 1.491 (95% HPD = 0.054–3.241). Lastly, the 5′ and 3′ RHs have an average rate of 0.995 (95% HPD = 0.054–3.235), and 1.209 (95% HPD = 0.054–3.209), respectively. All of these results are consistent with previous findings (Richard et al. 2015), with the *env_cenSU_* region apparently evolving much more rapidly than the rest of the *env* gene, 1.825 times faster on average (95% HPD on the linear scale = 0.045–60.423; log scale = −3.093 to 4.101).

### 2.3 Phylogenetic analysis

**Figure 2.**
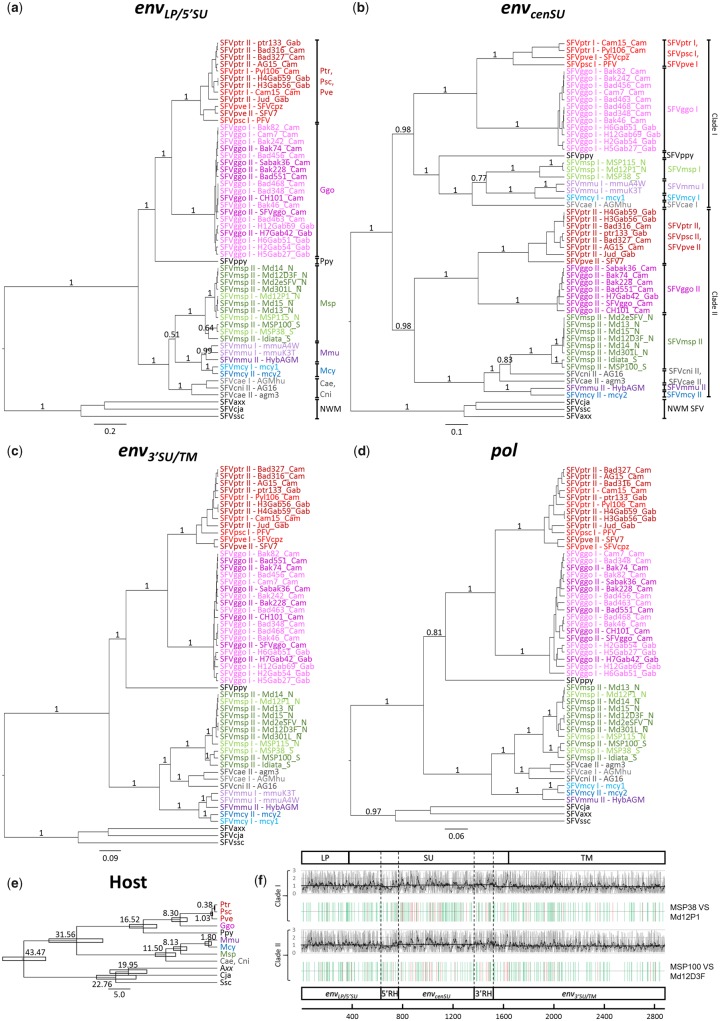
*env* and *pol* phylogenies. Bayesian phylogenies were estimated from (a) the *env_LP/5′SU_* alignment, (b) the *env_cenSU_* alignment, (c) the *env_3′SU/TM_* alignment, and (d) the *pol* alignment, which were 630, 600, 1,362, and 425 nt long, respectively. Numbers on branches are Bayesian posterior probability clade support values. Scale bars are in units of substitutions per site. For *env_LP/5_*_′__*SU*_, *env_3_*_′__*SU/TM*_, and *pol* phylogenies, sequences segregate according to their host species, forming monophyletic clades. In *env_cenSU_* phylogeny, OWMA FVs form two well-supported distinct clades, clades I and II. In addition to the label ‘I’ and ‘II’ in their names, light and darker colours indicate if sequences belong to clade I or II, respectively. (e) Host phylogeny. The topology of the host tree and the divergence dates were estimated elsewhere (see the references in Supplementary Table S2). (f) Site-wise evolutionary rate estimates of the *env* gene for variant I (top), and variant II (bottom). See legend to Fig. 1. The figure also shows the distributions of silent (green) and non-silent (red) substitutions, computed by using Highlighter (https://www.hiv.lanl.gov/content/sequence/HIGHLIGHT/highlighter_top.html): top, comparison between clade I SFVmsp MSP38 and SFVmsp Md12P1; bottom, between clade II SFVmsp MSP100 and SFVmsp Md12D3F. The distributions are nearly uniform. SFV, simian foamy virus; Psc, *Pan troglodytes schweinfurthii* chimpanzee (red); Pve, *Pan troglodytes verus* chimpanzee (red); Ptr, *Pan troglodytes troglodytes* chimpanzee (red); Ggo, *Gorilla gorilla gorilla* gorilla (pink); Ppy, *Pongo pygmaeus* orangutan (black); Msp, *Mandrillus sphinx* mandrill (green); Mmu, *Macaca mulatta* macaque (purple); Mcy, *Macaca cyclopis* macaque (blue); Cae, *Chlorocebus aethiops* Grivet (grey); Cni, *Cercopithecus nictitans* Greater spot-nosed monkey (grey); NWM, New World Monkey (black); A*xx*, *Ateles* spider monkey (black); Cja, *Callithrix jacchus* marmoset (black); Scc, *Saimiri sciureus* squirrel monkey (black).

Phylogenies of *env_LP/5_*_′__*SU*_, *env_cenSU_*, *env_3_*_′__*SU/TM*_, and *pol* were reconstructed separately under the Bayesian framework (Fig. 2). This accommodates and allows for potentially different evolutionary histories and dynamics of each genomic region. The results from the approximately unbiased (AU) tests (Table 2) confirmed that *env_LP/5_*_′__*SU*_, *env_cenSU_*, and *env_3_*_′__*SU/TM*_ have significantly different evolutionary histories. Thus, segments were analysed separately.

**Table 2.** AU tests. An AU test evaluates whether an inferred tree is supported by an alignment or not (Shimodaira 2002). An AU test P value of <0.05 suggests that the inferred tree is not supported by the alignment.

Alignment	AU test P value		
	*env_LP/5_*_′__*SU*_ phylogeny	*env_cenSU_* phylogeny	*env_3_*_′__*SU/TM*_ phylogeny
*env_LP/5_*_′__*SU*_	1.000	6 × 10^−7^	3 × 10^−5^
*env_cenSU_*	6 × 10^−29^	1.000	6 × 10^−4^
*env_3_*_′__*SU/TM*_	3 × 10^−5^	1 × 10^−3^	1.000

Analyses of *env_LP/5_*_′__*SU*_, *env_3_*_′__*SU/TM*_, and *pol* yielded similar results (Fig. 2a, c, and d), consistent with the well-established notion of FV–host co-speciation history (see the host evolutionary history in Fig. 2e). We found that sequences isolated from the same host species cluster together. Chimpanzee, gorilla, and orangutan-specific SFVs form monophyletic clades, with the clade of chimpanzee SFVs exhibiting a sister taxon relationship with that of gorilla SFVs, reflecting the host branching pattern. Chimpanzee SFV sequences further split into three subgroups, namely those of SFVptr, SFVpsc, and SFVpve, paralleling the host diversification. Old World monkey and ape FVs (OWMA SFVs) form a clade, which is in turn sister to the clade of New World monkey FVs (NWM SFVs), also mirroring the host evolutionary history. Moreover, we also found that sequences further segregate by the location of their host, in line with the FV–host co-speciation scenario: for example, SFVmsp sequences perfectly split into two phylogenetically distinct, well supported, groups—those of the North and the South of the Ogooué River.

This FV–host co-speciation pattern, although very strong, is nevertheless not absolute. A conflict between the branching orders of Old World monkeys and their FVs was observed, involving mandrill, Cercopithecini monkey, and macaque-specific SFVs. While the mandrill is more closely related to the macaque than the Cercopithecini monkey (Perelman et al. 2011), SFVmsp is more closely related to SFVcae and SFVcni than SFVmmu and SFVmcy. This result is strongly supported in *env_3_*_′__*SU/TM*_ and *pol* analyses (Bayesian posterior probability >0.99). Although analysis of *env_LP/5_*_′__*SU*_ showed that SFVmsp is more closely related to SFVmmu and SFVmcy than SFVcae and SFVcni, following the host diversification orders, this result is not well supported (Bayesian posterior probability = 0.51).

Analyses of *env_cenSU_*, in contrast, revealed a complex history for the *env* gene (Fig. 2b). We found that *env_cenSU_* sequences of OWMA SFVs segregate into two clades, clades I and II, each containing sequences of FVs isolated from chimpanzees, gorillas, Cercopithecini monkeys, and macaques, as well as those of mandrills reported in this study. This finding suggests that there are two variants of FVs that are cocirculating among OWMA populations, consistent with previous findings (Richard et al. 2015). Each clade exhibits some evolutionary features that are consistent with the notion of stable FV–host co-evolution. For example, in both clades, we could see that sequences obtained from the same host species cluster together, that OWMA SFVs form a clade to the exclusion of ape SFVs, and that the sequences also group by their host geographical location to some degree. Again, our analysis showed that in both of the clades *env_cenSU_* sequences of SFVmsp collected from the North of the Ogooué River are phylogenetic distinct from those collected from the South of the river. A number of FV–host evolutionary conflicts could also be observed within these two clades nonetheless. The results show that in clade II, SFVmsp is not sister to SFVmmu and SFVmcy, conflicting with the host speciation pattern, but mirroring the branching orders of the *env_3_*_′__*SU/TM*_ and *pol* phylogenies. The *env_cenSU_* sequence of the only SFVppy included in this study also appears to be more closely related to those of OWM SFVs, and not those of the ape SFVs.

This finding raised a particular concern regarding the observed elevated evolutionary rate of *env_cenSU_* (Fig. 1). Specifically, it could truly reflect an elevated rate of the *env_cenSU_* region, or alternatively, it could be an artefact of analysing the two *env* variants together. When site-wise rates were estimated exclusively for variant I sequences, we found that the average rates of different regions were comparable to each other (*env_LP/5_*_′__*SU*_: mean = 1.039, 95% HPD = 0.072–2.910; 5′ RH: mean = 0.895, 95% HPD = 0.072–2.965; *env_cenSU_*: mean = 1.131, 95% HPD = 0.072–2.954; 3′RH: mean = 1.162, 95% HPD = 0.072–2.874; *env_3_*_′__*SU/TM*_: mean = 0.916, 95% HPD = 0.072–2.833; Fig. 2f), and similar results were obtained for variant II strains (*env_LP/5_*_′__*SU*_: mean = 1.055, 95% HPD = 0.092–2.891; 5′RH: mean = 0.917, 95% HPD = 0.092–2.886; *env_cenSU_*: mean = 1.106, 95% HPD = 0.092–2.914; 3′RH: mean = 1.154, 95% HPD = 0.092–2.918; *env_3_*_′__*SU/TM*_: mean = 0.919, 95% HPD = 0.092–2.816; Fig. 2f). Indeed, the appearance of the elevated rate of evolution of *env_cenSU_* disappeared almost completely when the variant type was controlled for in the sequence comparison, 1.112 times faster on average than the rest of the gene for variant I sequences (95% HPD on the linear scale = 0.039–26.832; log scale = −3.251 to 3.290), and 1.052 times for variant II sequences (95% HPD on the linear scale = 0.045–22.572; log scale = −3.111 to 3.117). Consistent with these results are the near uniform distributions of the substitutions across the entire *env* gene for both variants I and II, obtained by comparing full *env* gene sequences of SFVmsp of the same variants (Fig. 2f). Combined, these results suggest that the observed elevated rate is likely an artefact of analysing the two *env* variants together.

Finally, another important observation is that the distinction between the two FV variants cannot be observed at all in analyses of *env_LP/5_*_′__*SU*_, *env_3_*_′__*SU/TM*_, and *pol* genes, with viruses from the two *env_cenSU_* clades are intermixing with one another. In other words, apart from this specific variant region of *env_cenSU_*, FVs of the same host species are not phylogenetically distinct.

### 2.4 Evolutionary timescales of the two *env* variants and the two SFVmsp populations

The co-evolutionary history between FVs and their mammalian hosts using polymerase genes or proteins have been repeatedly shown to be extremely stable, with the two co-diversifying with one another since the origin of eutherians, dated ∼100 Mya (Katzourakis et al. 2009, 2014). It was also reported that the rates of evolution of viruses, including those of FVs, appear to be increasingly slower the further we look back in time (Aiewsakun and Katzourakis 2016). This phenomenon, the so-called TDRP, can be described well by a simple power-law decay function (Aiewsakun and Katzourakis 2015). This, in turn, allows us to calibrate the timescale of the *env_cenSU_* phylogeny by converting its node heights from the units of substitutions per site (s estimates) to time (t estimates) by using the equation $\log\left(t\right)=\alpha +\beta \log\left(s\right)$. This method has been shown to be more accurate than the current relaxed clock models for FV rate estimation, and more generally where the TDRP has been in effect, and the number of calibration points is limited (Aiewsakun and Katzourakis 2015).

To compute the TDRP model parameters, α and β, calibration dates are required. Given the complex evolutionary history of *env_cenSU_*, only three events of SFV divergence could be mapped confidently onto those of their hosts based on the tree topology comparison: 1, the divergence between chimpanzee and gorilla SFVs in clades I and II, 2, the divergence between SFVmcy and SFVmmu in clades I and II, and 3, the divergence between OWMA SFVs and NWM SFVs. Previous analysis of *pol* sequences, however, showed that the evolutionary rate dynamics of NWM SFVs might differ from those of OWMA SFVs (Aiewsakun and Katzourakis 2015). Although our analysis does not involve *pol* sequences, we nevertheless decided not to include the latter data point in the TDRP model estimation to avoid any potential errors. By doing so, this left us with only two independent data points for the calibration of the model, which contains two parameters. This could cause overfitting and the resultant model might have poor predictive power. Many studies have suggested that the separation between SFVs and fereungulata FVs, including bovine, equine, and feline FVs (BFV, EFV, and FFV, respectively), is a co-speciation event, with BFV and EFV being more closely related to one another than FFV (Bininda-Emonds et al. 2007; Katzourakis et al. 2009, 2014). To estimate the TDRP model parameters, α and β, we re-estimated the *env_cenSU_* phylogeny with the inclusion of BFV, EFV, and FFV, and used the node height corresponding to their separation from SFVs to calibrate our TDRP model in addition to the two aforementioned data points.

**Figure 3.**
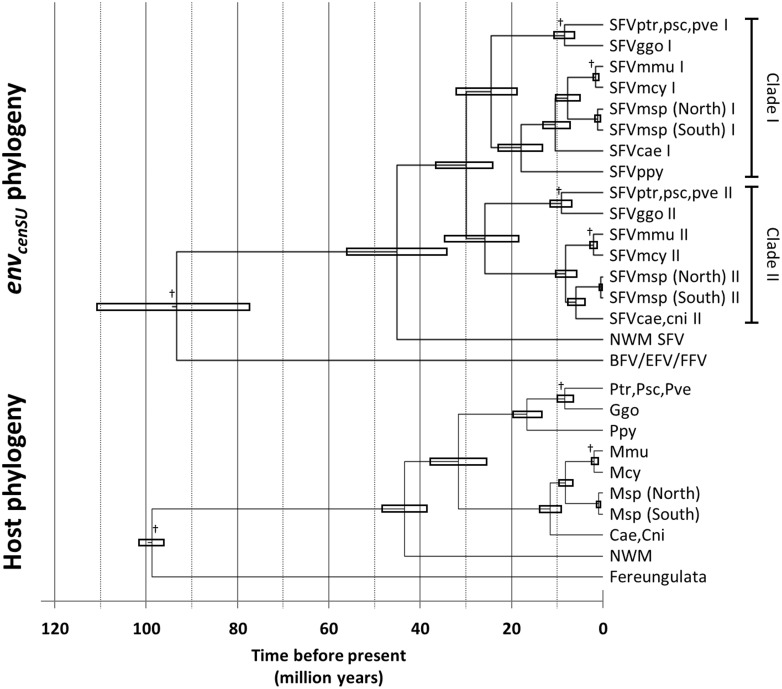
Time-calibrated *env_cenSU_* (top) and host (bottom) phylogenies. The timescale of the *env_cenSU_* phylogeny was calculated under the TDRP model: $\log\left(t\right)=\alpha +\beta \log\left(s\right)$, where t and s are time, and the corresponding number of substitutions per site, respectively, with the model parameter α = 4.114 (95% HPD = 3.827–4.383), β = 1.762 (95% HPD = 1.556–1.985). Sequences from fereungulata FVs were included to better estimate the TDRP model. The tree was drawn manually as the TDRP model has not yet been implemented in BEAST, and shows only nodes with important timescales we discussed in the study. The topology of the host tree and the divergence dates were estimated elsewhere (see the references in Supplementary Table S2). The node bars represent the uncertainties of the evolutionary timescale. The time is in the units of million years. The symbol ‘†’ denotes the nodes used in the TDRP model calibration. The estimated divergence dates of viruses and their hosts, as well as associated uncertainties, can be found in Supplementary Table S2. SFV, simian foamy virus; Psc, *Pan troglodytes schweinfurthii* chimpanzee; Pve, *Pan troglodytes verus* chimpanzee; Ptr, *Pan troglodytes troglodytes* chimpanzee; Ggo, *Gorilla gorilla gorilla* gorilla; Ppy, *Pongo pygmaeus* orangutan; Mmu, *Macaca mulatta* macaque; Mcy, *Macaca cyclopis* macaque; Msp, *Mandrillus sphinx* mandrill; Cae, *Chlorocebus aethiops* grivet; Cni, *Cercopithecus nictitans* greater spot-nosed monkey; NWM, New World Monkey; BFV, bovine foamy virus; EFV, equine foamy virus; FFV, feline foamy virus.

Under the TDRP model, we estimated the origin of the two *env* variants to be ∼29.892 (95% HPD = 24.230–36.743) Mya, coinciding with the diversification of OWMAs, which happened ∼31.56 Mya (Perelman et al. 2011). As internal controls, we inferred the timescale of OWM SFVs and of SFVs as a whole using the estimated TDRP model, and found that they are comparable to those of their hosts for both SFV variants (Fig. 3 and Supplementary Table S2). This indicates that the FV–host co-speciation assumption we used to infer the TDRP model is self-consistent and is of a high predictive value.

In addition, we estimated the evolutionary timescale of SFVmsp to be ∼1.075 (95% HPD = 0.546–1.708), and ∼0.415 (95% HPD = 0.171–0.718) Myr for clades I and II, respectively (Fig. 3 and Supplementary Table S2). These results are comparable to those obtained from the analyses of *env_LP/5_*_′__*SU*_ (median = 1.141 Myr, 95% HPD = 0.379–2.176 Myr), *env_3_*_′__*SU/TM*_ (median = 0.984 Myr, 95% HPD = 0.459–1.618 Myr), and *pol* (median = 1.737 Myr, 95% HPD = 0.698–2.889 Myr) (Table 3). This again indicates that our analyses are all consistent with one another and suggests the two populations of mandrills were separated from each other by the river about 1 Mya. These estimates also match the host divergence date, estimated to be ∼0.80 (0.56–1.40) Mya based on the *cytochrome b* gene (Telfer et al. 2003). Combined with the observed complete phylogenetic separation of the North and South SFVmsp sequences, these results strongly support that SFVmsp co-diversifies with their hosts.

**Table 3.** Evolutionary timescales of mandrill-speciation simian foamy viruses. The timescales were estimated under the FV–host co-speciation assumption by using the TDRP models: $\log\left(t\right)=\alpha +\beta \log\left(s\right)$; where t and s are time, and the corresponding number of substitutions per site, respectively. For *env_LP/5_*_′__*SU*_, α = 4.487 (95% HPD = 3.964–5.114), β = 1.746 (95% HPD = 1.377–2.217); for *env_cenSU_*, α = 4.114 (95% HPD = 3.827–4.383), β = 1.762 (95% HPD = 1.556–1.985); for *env_3_*_′__*SU/TM*_, α = 4.522 (95% HPD = 4.098–4.973), β = 1.553 (95% HPD = 1.273–1.892); and for *pol*, α = 4.681 (95% HPD = 4.100–5.320), β = 1.419 (95% HPD = 1.093–1.820). See Section 5 for the nodes used in the TDRP model calibrations.

Genomic sequence	Evolutionary timescale (million years)		
	Median	Upper bound 95% HPD	Lower bound 95% HPD
*env_LP/5_*_′__*SU*_	1.141	0.379	2.176
*env_cenSU_* clade I	1.075	0.546	1.708
*env_cenSU_* clade II	0.415	0.171	0.718
*env_3_*_′__*SU/TM*_	0.984	0.459	1.618
*pol (integrase)*	1.737	0.698	2.889

Overall, our results suggest that both SFV variants have been co-evolving in a stable fashion with their hosts since the origin of OWMAs.

### 2.5 Comparison of the N-glycosylation patterns of the two *env* variants

N-glycosylation plays important roles in determining biological and molecular properties of the SFV Env protein (Luftenegger et al. 2005), including the ability to bind to cell receptors (Duda et al. 2006). Since the *env_cenSU_* region spans the RBD, we examined if the N-glycosylation patterns were different between the two *env* variants.

**Figure 4.**
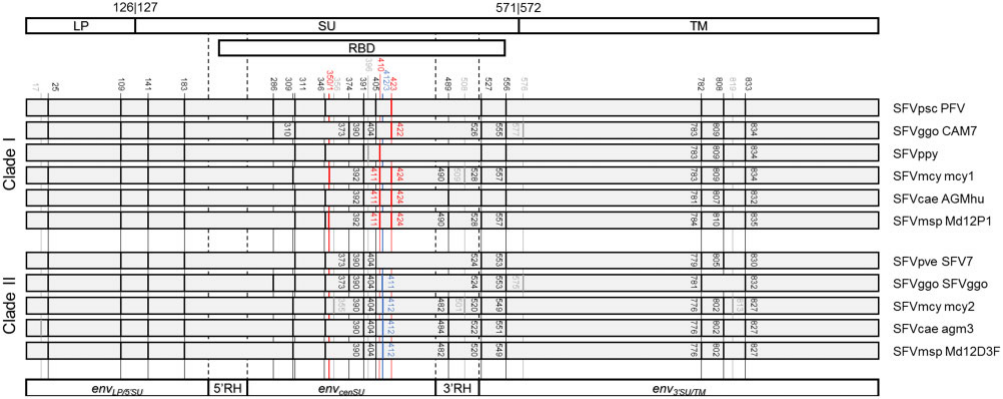
N-glycosylation sites on the Env proteins. Top, schematic outline of the Env domain structure. The numbers shown indicate the beginning and the end of the domains with respective to that of the PFV (accession number: CAA69004, reported in the GenBank file Y07725) (Duda et al. 2006). Middle, N-glycosylation pattern on SFV Env proteins. N-glycosylation sites were predicted using NetNGlyc 1.0 server (http://www.cbs.dtu.dk/services/NetNGlyc/) for eleven sequences. Six are those belonging to clade I, and five are those in Clade II. Sites that differ by only one amino acid are annotated together. Black lines indicate those that are shared between the two variants. Red lines indicate those that are present only in variant I sequences, and blue lines mark sites that are unique to variant II sequences. N-glycosylation sites that are unique to one host-specific viral group are marked with grey lines. The numbers are the locations of the predicted N-glycosylation sites in each protein sequence. Bottom, schematic outline of the *env_LP/5_*_′__*SU*_, *env_cenSU_*, *enu_3′SU/TM_*, and RH regions of the *env* gene. LP, leader peptide; SU, surface domain; TM, transmembrane domain; RBD, receptor binding domain; RH, recombination hotspot; SFV, simian foamy virus; Psc, *Pan troglodytes schweinfurthii* chimpanzee; Pve, *Pan troglodytes verus* chimpanzee; Ggo, *Gorilla gorilla gorilla* gorilla; Ppy, *Pongo pygmaeus* orangutan; Mcy, *Macaca cyclopis* macaque; Cae, *Chlorocebus aethiops* grivet; Msp, *Mandrillus sphinx* mandrill.

N-glycosylation sites were predicted for eleven sequences by using the NetNGlyc 1.0 server (http://www.cbs.dtu.dk/services/NetNGlyc/) (Fig. 4). Six were those of variant I, including sequences from chimpanzee, gorilla, orangutan, macaque, African green monkey, and mandrill-specific SFVs. The rest were those of variant II, including sequences from chimpanzee, gorilla, macaque, African green monkey, and mandrill-specific SFVs. The known fifteen N-glycosylation sites in PFV (Luftenegger et al. 2005) were recovered, suggesting that the prediction is effective. The majority of the predicted N-glycosylation sites were shared between the two variants. Nevertheless, three sites, which were located in the RBD mapping to aa 350/1, 410 and 423 of the PFV Env protein (accession number: CAA69004, reported in the GenBank file Y07725), are unique to variant I, and are shared among SFVs of different host species. Furthermore, we also identified one unique site for variant II, mapping to aa 412/3 of the PFV Env protein (accession number: CAA69004). These findings suggest that the two *env* variants might allow viruses to interact with different host receptors.

## 3. Discussion

We report the cocirculation of two *env* variants in mandrill populations in Central Africa. We determined that the RBD, located within the SU flanked by two RHs, which we termed the *env_cenSU_* region, defines the two *env* variants (Fig. 1). Recombination of *env* genes is not unique to SFVs, and has been observed in other strains of FVs, including feline FVs (Winkler et al. 1998; Phung et al. 2001), and also various other replication-competent retroviruses, including human immunodeficiency viruses (HIVs). An intra-host evolutionary study, for example, identified the HIV-1 *env* gene to harbour RHs around the surface-exposing variable regions (Song et al. 2018), which our results mirror.

Previous studies have shown that *env_cenSU_* appears to evolve faster than the rest of the *env* gene (Galvin et al. 2013; Richard et al. 2015), and accordingly it was termed a ‘variant’ region, while the rest of the *env* gene was termed a ‘conserved’ region (Richard et al. 2015). Our analysis produced similar results (Fig. 1). However, when the site-wise evolutionary rates were computed separately for each variant, the effect disappeared almost completely (Fig. 2f). Our results thus suggest that the rate of *env_cenSU_* evolution is comparable, or only slightly higher, than the rest of the *env* gene, and that the observed elevated rate was largely due to the two *env* variants having been analysed together.

Phylogenetic analyses revealed that different parts of the SFV genome have drastically different evolutionary histories. Phylogenetic analyses of *env_LP/5_*_′__*SU*_, *env_3_*_′__*SU/TM*_, and the integrase coding domain (*pol*) yielded similar results (Fig. 2). Both show that SFVs isolated from the same host species cluster together, and their branching orders mostly parallel those of their hosts. Furthermore, as previously noted, segregation of SFVs by host geography could be found in the clade of SFVggo, in which the Cameroonian and Gabonese strains form two distinct clades (Richard et al. 2015). The same pattern could also be observed in the clade of SFVmsp, in which the isolates obtained from the North and the South of the Ogooué River form two separate phylogenetic groups. It is possible that this perfect separation of the North and the South SFVmsp could occur by chance due to our small sample size (seven northern SFVmsp sequences, and three southern SFVmsp sequences, Table 1). Nevertheless, it has been demonstrated with larger datasets that the Ogooué River is indeed a major gene-flow barrier that can lead to the diversification of SFVmsp (Telfer et al. 2003; Mouinga-Ondeme et al. 2010) and mandrill monkeys (Telfer et al. 2003), separating both hosts and viruses into two phylogenetically distinct populations. Similar observations have also been reported for simian immunodeficiency virus (Souquiere et al. 2001), and simian T-cell leukemia virus infecting mandrills (Makuwa et al. 2004). Our results are comparable to these previous findings, and we believe that they truly reflect geographical isolation of the North and South Gabon SFVmsp. Furthermore, our analyses of these three genomic regions estimated their divergence dates to be between 0.984 and 1.737 Mya (*env_LP/5_*_′__*SU*_: median = 1.141 Mya, 95% HPD = 0.379–2.176 Mya; *env_3_*_′__*SU/TM*_: median = 0.984 Mya, 95% HPD = 0.459–1.618 Mya; and *pol*: median = 1.737 Mya, 95% HPD = 0.698–2.889 Mya; Table 3), coinciding with the host divergence date, estimated to be ∼0.80 (0.56–1.40) Mya (Telfer et al. 2003). All these observations and results are consistent with the notion of stable virus–host co-speciation.

However, analysis of the *env_cenSU_* region revealed two subclades of OWMA SFVs for each of the host-specific groups, indicative of the cocirculation of two variants of SFVs within OWMA populations (Richard et al. 2015). Similar to the results obtained from analyses of *env_LP/5_*_′__*SU*_, *env_3_*_′__*SU/TM*_, and *pol* sequences, for each of the two variants, we found that sequences segregate according to their host species, forming host-specific monophyletic clades. They also group by their host geographical locations to some extent. Again, our analysis shows that, for both variants, SFVmsp isolates collected from the North of the Ogooué River cluster together, and they are phylogenetically distinct from those collected from the South of the river. The divergence dates of the two populations were estimated to be ∼1.075 (95% HPD = 0.546–1.708), and ∼0.415 (95% HPD = 0.171–0.718) Mya for clades I and II, respectively (Fig. 3), similar to those estimated for other genomic regions (Table 3) and that of their hosts (Telfer et al. 2003). Since some of our viruses were isolated from captive animals (Table 1), we have to consider the possibility that the existence of the two virus variants in both North and South mandrill populations could be due to viral transmission during captivity. However, since the three mandrills from the southern area are wild animals (Table 1), our analyses thus conclusively show that both variants do exist in nature, at least in the southern area. Also, our analyses showed that, for both variants, the North and South SFVmsp populations diverged at least hundreds of thousands of years ago. This eliminates the possibility that the observed northern mandrill SFVs were a result of transmission from the southern mandrills in captivity, and at the same time further supports that both variants do indeed exist and cocirculate in the natural northern and southern mandrill populations, co-diversifying with their hosts. Furthermore, we also estimated the origin of the two *env* variants to coincide with the diversification of OWMAs (Fig. 3). Together, our results strongly support that both SFV variants have been cocirculating in the wild host populations and co-evolving in a stable fashion with their hosts since the origin of OWMAs.

Despite the fact that both of the *env_cenSU_* variants have a stable co-evolutionary history with their hosts, with each host-specific group forming two defined distinct clades, analyses of the *env_LP/5_*_′__*SU*_ region, *env_3_*_′__*SU/TM*_ region, and the *pol* gene, could not recover such a pattern, but instead show that the two SFV variants intermix with one another. This surprising result is indicative of a modular evolutionary nature of the SFV *env* gene. Three evolutionary mechanisms are proposed to explain this observation:

One possibility is that there are two strains of OWMA SFVs that have been co-diversifying with their hosts over the past 30 Myr, and evolve independently from one another. However, each strain experiences an extreme convergent evolutionary pressure across the entire genome such that they are phylogenetically indistinguishable except only for the *env_cenSU_* region, perhaps due to host cell-receptor adaptation. This evolutionary scenario could explain the co-speciation pattern observed in each of the two clades of OWMA *env_cenSU_*, and the intermixing of the two variants for the rest of the genome without invoking recombination. However, we find it hard to imagine that there exists such an extreme convergent evolutionary pressure across almost the entire genome apart from one region, and believe that this is unlikely to be the case.

Another possibility is that there is a ‘ghost virus’ lineage, which has yet to be sampled. It is important to note that, since both variants of *env_cenSU_* are ancient in origin, being as old as their OWMA hosts, their ultimate source cannot be modern-day SFVs, but an ancient virus. This ancient ghost lineage would have been cocirculating with SFVs in OWMA populations over the past ∼30 Myr, and this has led to recombination between SFVs’ *env* genes and those of the ghost lineage, giving rise to the two (apparent) SFV variants as defined by the *env_cenSU_* recombinant region. Indeed, this hypothesis has already been proposed in previous studies (Galvin et al. 2013; Richard et al. 2015). Our data do not suggest which variant is the recombinant strain or the core strain however, as the two *env* variants are present in similar quantities in all host species, exhibit similar evolutionary patterns, and form separate monophyletic clades, of which the branching patterns (Fig. 2) and dates (Fig. 3) largely mirror those of their hosts. Furthermore, to explain the co-diversification pattern observed in both OWMA *env_cenSU_* clades, this mechanism suggests that the ghost viruses likely co-diversify with their OWMA hosts, under the assumption that the evolutionary history of *env_cenSU_* reflects that of the rest of the genome. Moreover, if the recombination were to happen only once and the recombinants were to survive until today, then the rest of the genome should have formed two distinct clades, but this is not the case. Instead, we found that the rest of the genome of the two SFV variants is phylogenetically indistinguishable. This means that the recombinants do not survive for very long, and that the recombination must have happened multiple times with great frequency and relatively recently. Combining these observations, this ghost virus lineage hypothesis implies that the ghost viruses are present in all of the investigated OWMA populations, co-diversify with their hosts, and recombine with the other SFVs. It also implies that the recombinants die young, but are continually resupplied due to frequent recombination.

The natural follow-up question is then; what are these elusive ancient ghost viruses? Could it be an ancient endogenous virus, which potentially could explain frequent recombination and the long-term co-speciation patterns that we observed in both clades? We searched various NCBI nucleotide/genomic databases by using tBLASTn with Env_cenSU_ protein sequences as probes, but the search only returned FV sequences. This result suggests that the source of genetic variation is unlikely to be endogenous viruses, and that the ghost viruses are perhaps SFVs. However, if they were to truly be another ancient lineage of OWMA SFVs, this hypothesis would imply that in all OWMA populations there exist two distinct lineages of endemic SFVs that diverged around 30 Mya and both have been independently co-diversifying with their hosts since then. Nevertheless, every effort to sequence FVs so far has only ever resulted in the discovery of one specific lineage and not the other, since, to the best of our knowledge, there are no studies reporting the discovery of two distinct endemic SFV lineages in one host species, except for those identified by analyses of *env* genes (Galvin et al. 2013; Richard et al. 2015). Another possibility is that the ghost viruses might not be FV-like at all. This, however would imply that the *env* genes of the non foamy-like ghost viruses are under such strong selection pressure that they had evolved to become FV-like. Given the assumptions and consequences of this evolutionary scenario, we believe that it is also unlikely.

The third possible evolutionary mechanism is that there are two strains of OWMA SFVs as defined by *env_cenSU_*, and they have been cocirculating within the same host populations over the past 30 Myr, with each strain co-diversifying with their hosts. Nevertheless, these two strains do not evolve independently from one other, but frequently interchange their *env_cenSU_* domains via recombination. This mechanism allows *env_cenSU_* to form two divergent clades, each exhibiting separate co-speciation patterns with their hosts, while the rest of the genome evolves separately as if it is of a single virus lineage, and again with its own virus–host co-diversification history. This hypothesis predicts specifically that, since both variants of the *env_cenSU_* region belong to the same gene pool, they should have similar codon usages (while this might not be true for the ‘ghost lineage’ hypothesis, e.g.). Comparison of their codon usages, including that of the *env_LP/5_*_′__*SU*_, and *env_3_*_′__*SU/TM*_ regions combined, showed that they are not significantly different (χ^2^ test: χ^2^ = 6.546, degrees of freedom = 120, P value = 1), consistent with this hypothesis. Of the three hypotheses, this is perhaps the simplest and less extraordinary one in term of assumptions and consequences.

Although the origin of these two *env* variants is still unclear, our results beg the question: what could possibly maintain these two *env* molecular variants in all of these separate OWMA populations over the past 30 Myr? One possible answer is that, as previously discussed in Richard et al. (2015), these two SFV variants may utilize the two different host receptor proteins, one with high and the other with low affinity (Herchenroder et al. 1999). This is supported by the observations that the *env_cenSU_* recombinant region maps precisely to the RBD, and that the N-glycosylation patterns are different between the two variants, but which are common among those of the same variant (Fig. 4). Indeed, it has been shown in HIV that recombination of *env* genes can lead to immune escape (Streeck et al. 2008), and changes in cell tropisms (Mild et al. 2007; Brown et al. 2011; Nishimura et al. 2011), suggesting that this might be possible.

## 4. Conclusion

This study reports two phylogenetically distinct populations of SFVmsp which are separated geographically by the Ogooué River into the North and the South Gabon populations, co-diversifying with their hosts. We show that SFVmsp has two variants of *env* genes that co-exist with one another differing in their SUs (*env_cenSU_*), and they can be found in both of the two SFVmsp populations, as reported for other SFVs, including those of chimpanzee, gorilla, macaque, and Cercopithecini monkey (Galvin et al. 2013; Richard et al. 2015). We estimated that the two *env* variants emerged ∼30 Mya, matching the time during which OWMAs started to diversify.

Surprisingly, our analyses show that the SFV SU has an evolutionary trajectory entirely separate from the rest of the genome. While most of the genome evolves as a single virus lineage, the SU variants evolve independently and are maintained in all separate OWMAs. Indeed, recombination of SFV *gag* (Engel et al. 2013; Feeroz et al. 2013) and *pol* (Liu et al. 2008) genes have been observed. However, we note that, to the best of our knowledge, all previously reported recombination events, specifically those observed in SFV *gag* and *pol* genes, were sporadic, short-lived, and were of small scale, occurring within individual hosts or populations, and were between closely related SFV strains. Our study, however, has revealed systemic and independent recombination events of a single region located within the *env* gene across a wide range of different host-specific SFV groups. Our analyses showed that, although the recombinant strains are not stable, the two *env_cenSU_* variants corresponding to the recombinant region themselves co-evolve largely independently from the rest of the genome for the past 30 Myr. To the best of our knowledge, no other studies have reported such results for SFV *gag* and *pol* genes.

Three possible evolutionary mechanisms are considered to explain this observation: 1, extreme convergence of the entire genome excluding the RBD, 2, very frequent, recent, and extremely wide spread recombination between SFVs’ *env* genes and the *env* genes of an unknown ancient ghost virus in a wide range of OWMAs, or 3, SFVs having two versions of *env* genes that stably co-exist with one another and can ‘switch places’ so readily that they effectively allow the rest of the genome to evolve as one virus lineage. While these hypotheses sound improbable, they are consistent with, if not required to explain, our observations. Considering their implications, we believe that the last mechanism is the most likely one.

Although our results imply very widespread, and presumably very frequent, recombination among SFVs (and perhaps with other viruses), they nevertheless still support the notion of the long-term and stable FV–host co-speciation history. This work highlights the modular nature of FV genomes, and shows that their evolutionary dynamics and interactions with their hosts and among each other might be much more complex than previously thought.

## 5. Materials and methods

### 5.1 SFV-infected mandrill DNA samples collection

In three previous studies, SFV-infected mandrills had been identified (Calattini et al. 2004; Mouinga-Ondeme et al. 2010, 2012). These animals were either from a semi-free ranging colony in the Primatology Centre of the International Centre for Medical Research in Franceville (Gabon), or wild-born and wild-caught animals (Gabon and Cameroon). Blood samples had been collected (in accordance with the rules of animal care committees) into EDTA tubes. The buffy coat had been obtained after centrifugation and genomic DNA extracted by using the QIAamp DNA blood minikit (Qiagen, Courtaboeuf, France). SFV-infection had been assessed by serological screening and a 465-bp-long *pol-integrase* PCR. At the beginning of the study, eleven DNA samples from SFV-infected mandrills were available (Table 1). To ensure good quality, DNA samples were subjected to a β-globin PCR. Moreover, SFV status was confirmed through the *pol-integrase* PCR (Calattini et al. 2004; Mouinga-Ondeme et al. 2010, 2012).

### 5.2 PCR amplification of *env* from genomic DNA

No SFVmsp *env* sequence was available at the beginning of the study. Thus, degenerate PCR primers were designed by targeting mostly conserved regions of the *env* gene of all SFV available in literature (Supplementary Table S1). The complete SFV *env* sequences were obtained through five overlapping nested PCRs.

Nested PCRs were performed as follows: 500 ng of DNA was mixed in the enzyme buffer with the external primers (0.25 µM each), MgCL_2_ (3.5 mM), deoxynucleoside triphosphates (dNTPs) (200 µM each) and 0.5 µl of HotStarTaq polymerase (Qiagen) in a final volume of 50 µl. External PCR consisted of a 15-min-long denaturation step at 95 °C, followed by 40 amplification cycles (40 s at 95 °C, 40 s at 50 °C, and 1 min per kb at 72 °C) and a 7-min-long extension step at 72 °C. The product (5 µl) was then used as template for a second internal PCR, under the same conditions by using internal primers. PCR products were directly sequenced by MWG operon (Ebersberg, Germany). Both sense and antisense sequences were obtained for each fragment and were found identical. To obtain complete *env* sequences, the different *env* fragments were concatenated.

### 5.3 *pol* and *env* nucleotide alignments

Fifty-one partial *pol* nucleotide sequences corresponding to the integrase coding region, and forty-seven complete *env* genes of SFVs were aligned in MEGA 7 (Kumar, Stecher, and Tamura 2016) by using MUSCLE (Edgar 2004) with default parameters. Seven additional partial *env* sequences corresponding to the SU-coding region were also included in the *env* alignment. After manual curation, the *pol* and *env* alignments were 425 and 2,883 nucleotides (nt) long, respectively. The alignments are available from the authors upon request.

### 5.4 Recombination detection

Potential recombination within the *pol* and *env* alignments was checked using seven programmes, including RDP, GENECONV, Chimaera, MaxChi, BootScan, SiScan, and 3Seq, implemented in Recombination Detection Program 4 (Martin et al. 2015) with their default settings. Recombination events detected by less than four programmes, at a P value <0.05, were not included in downstream analyses, and the plot of recombination event map was used to determine RHs. Under this setting, while recombination events could not be detected in the *pol* alignment, two RHs were found in the *env* alignment. The first hotspot lies approximately between 631 and 768 nt, and the second lies approximately between 1,369 and 1,521 nt of the alignment. Site-wise rates of *env* evolution were estimated by using MEGA 7 (Kumar, Stecher, and Tamura 2016) under the maximum likelihood framework with GTR + I + γ(4) nucleotide substitution model. All nucleotide positions were used in the analyses and the rates were scaled such that the average evolutionary rate across all sites is 1.

The *env* alignment was subsequently divided into three alignments, excluding of the two RH regions: 1, from 1 to 630 nt (*env_LP/5_*_′__*SU*_), 2, from 769 to 1,368 nt (*env_cenSU_*), and 3, from 1,522 to 2,883 nt (*env_3_*_′__*SU/TM*_) of the full *env* alignment. We noted that, while our analyses suggested that the *env* sequence ‘Bad316’ is a recombinant, its recombination break points lie outside the two RHs however, estimated to be at 481 and 2,130 nt. We thus removed its nucleotide sequence from 481 to 630 nt from the *env_LP/5_*_′__*SU*_ alignment, and from 1,522 to 2,130 nt from the *env_3_*_′__*SU/TM*_ alignment, otherwise they may bias downstream phylogenetic analyses. The alignments are available from the authors upon request.

### 5.5 Phylogenetic analysis

Bayesian phylogenies of the *pol*, *env_LP/5_*_′__*SU*_, *env_cenSU_*, and *env_3_*_′__*SU/TM*_ were estimated from their nucleotide alignments by using BEAST 1.8.4 (Drummond et al. 2012). The Yule speciation tree prior was selected because we were most interested in the macroevolutionary history and dynamics of SFVs of different host-specific groups. The strict molecular clock was applied and the total-per lineage substitution numbers to various internal nodes were collected for the purpose of estimating divergence dates of various SFV groups under the power-law rate decay model (see below). By using FVs as the subject of study, this model has been demonstrated to be more accurate than currently available relaxed molecular clocks where the TDRP is evident (Aiewsakun and Katzourakis 2015), and hence it was used in this study.

The best-fit nucleotide substitution models were used in the analyses (*pol*: TVM + γ(4), *env_LP/5_*_′__*SU*_: GTR + γ(4), *env_cenSU_*: TIM3 + I + γ(4), and *env_3_*_′__*SU/TM*_: GTR + I + γ(4)), determined by JModelTest 2.1.10 under the corrected Akaike information criterion (Darriba et al. 2012). The MCMC was run for 100,000,000 steps. The initial 10 per cent was discarded as burn-in, and the parameters were logged every 1,000 steps thereafter. Parameter value convergence and sampling independency were manually inspected with Tracer v1.6 (Rambaut, Suchard, and Drummond 2013). All parameters had an effective sample size of >5,200, indicating that all of them were well sampled and had converged.

AU tests were performed to investigate whether or not *env_LP/5_*_′__*SU*_, *env_cenSU_*, and *env_3_*_′__*SU/TM*_ have the same evolutionary history (Shimodaira 2002). The site-wise log likelihoods used in the tests were computed by using PAML 4.9e (Yang 2007). The calculation was performed with the GTR + γ(4) nucleotide substitution model, the best available model for all alignments determined by JModelTest 2.1.10 under the corrected Akaike information criterion (Darriba et al. 2012). A molecular clock was imposed, and ambiguous sites were included in the analyses. AU tests were performed in Consel with default parameter settings (Shimodaira and Hasegawa 2001).

### 5.6 Calibrating the evolutionary timescale of *env_cenSU_* phylogeny

Rates of evolution of viruses, including those of FVs, appear to be increasingly slower the further we look back in time (Aiewsakun and Katzourakis 2016). This phenomenon, the so-called TDRP, can be best described by a simple power-law decay function (Aiewsakun and Katzourakis 2015). One of the implications of the TDRP is that the relationship between total per-lineage substitution (s) and evolutionary timescale (t) follows this equation: $\log\left(t\right)=\alpha +\beta \log\left(s\right)$ (Aiewsakun and Katzourakis 2016).

Estimating the α and β parameters of the TDRP model necessitated corresponding t and s estimates, and these data could be obtained under the FV–host co-speciation assumption. We re-estimated the *env_cenSU_* phylogeny with the inclusion of fereungulata FVs, including BFV, EFV, and FFV (see Section 2). The alignment contains fifty-seven sequences and is 637 nt in length after the curation, and is available from the authors upon request. Three co-diversification events were inferred from its topology: 1, the split between chimpanzee and gorilla SFVs, 2, the divergence between SFVmcy and SFVmmu, and 3, the separation of SFVs and fereungulata FVs. Corresponding s and t estimates of these inferred events (Supplementary Table S2) were used to calibrate the TDRP model (see below).

The phylogeny was re-estimated under the Bayesian phylogenetic framework by using BEAST 1.8.4 (Drummond et al. 2012), with fereungulata FVs constrained to form a monophyletic clade and BFV constrained to be a sister taxon of EFV. The same parameter setting as described above was applied in this analysis with the TIM3 + I + γ(4) substitution model, determined to be best-fit to the data by JModelTest 2.1.10 under the corrected Akaike information criterion (Darriba et al. 2012). All parameters had an effective sample size of >4,700, indicating that they were well sampled and had converged.

To estimate the timescale of the *env_cenSU_* phylogeny, for each of the 9,000 sampled Bayesian posterior trees, we extracted the heights of the nodes (s estimates) of the three aforementioned FV–host co-speciation events. The corresponding timescales of the events (t estimates) were derived from those of their hosts. The t estimates were sampled from normal distributions, with the means equal to the median estimates reported in the literature, and their standard deviations derived from the reported upper- and lower-bounds of the corresponding 95 per cent HPD intervals: $\max\left(\mathrm{Median}-\mathrm{Lower}\;95\%\;\mathrm{HPD}\;\mathrm{limit}/1.96,\;\mathrm{Upper}\;95\%\;\mathrm{HPD}\mathrm{limit}-\mathrm{Median}/1.96\right)$. The s and t estimates were then log-transformed, and we in turn fitted a linear model to them. The calibrated model, $\log\left(t\right)=\alpha +\beta \log\left(s\right)$, was then used to compute the t estimates of other nodes given their s estimates. This process was applied to all of the 9,000 posterior estimated trees to obtain the full distributions of t estimates (Supplementary Table S2).

### 5.7 Evolutionary timescale of SFVmsp, estimated from *env_LP/5′SU_*, *env_3′SU/TM_*, and *pol* sequences

From the posterior distributions of *env_LP/5_*_′__*SU*_, *env_3_*_′__*SU/TM*_, and *pol* phylogenies that we inferred (see Sections 5 and 5.5), we extracted the node heights of the clade of SFVmsp, and subsequently used them to estimate the SFVmsp evolutionary timescale by using the TDRP models under the FV–host co-speciation assumption, as described above. However, unlike in the case of *env_cenSU_*, we were able to infer five SFV–host co-speciation events by comparing the host and the SFV *env_LP/5_*_′__*SU*_, *env_3_*_′__*SU/TM*_, and *pol* tree topologies. These included 1) the divergence of chimpanzee and gorilla SFVs, 2) the divergence of orangutan-specific SFV (SFVppy) from the clade of chimpanzee and gorilla SFVs, 3) the separation of SFVmcy from SFVmmu, 4) the basal diversification of OWM SFVs, and 5) the basal diversification of OWMA SFVs. As a result, fereungulata FVs were not needed in this analysis.

### 5.8 N-glycosylation site prediction

N-glycosylation patterns were predicted for eleven sequences, six from clade I and five from clade II (Fig. 4), by using NetNGlyc 1.0 server (http://www.cbs.dtu.dk/services/NetNGlyc/). The known fifteen N-glycosylation sites in PFV were recovered (Luftenegger et al. 2005), suggesting that the prediction is effective.

### 5.9 Codon usage comparison

Codon frequencies were estimated for three datasets by using MEGA 7 (Kumar, Stecher, and Tamura 2016). This includes 1, the *env_cenSU_* region of sequences in clade I, 2, the *env_cenSU_* region of sequences in clade II, and 3, the *env_LP/5_*_′__*SU*_, and *env_3_*_′__*SU/TM*_ regions combined. Their codon frequencies were compared using the χ^2^ test. See Supplementary Table S3 for the codon frequency table.

## Author contributions

P.A., A.G., P.V.A., and A.K. conceived the project. L.R., A.G., A.M.O., P.V.A. collected the samples and performed the molecular work. P.A., A.K. performed the analyses and wrote the paper. All authors read and approved the manuscript.

## Funding

This study received funding from the CNRS (UMR 3569), the Institut Pasteur, France, the ‘Centre international de Recherches Médicales de Franceville’ (CIRMF) in Gabon and through the ‘Investissement d’Avenir’ as part of a ‘Laboratoire d’Excellence’ (LabEx) French research programme: Integrative Biology of Emerging Infectious Diseases (ANR10-LBX-62 IBEID) (A.G.). Léa Richard was financially supported by the ANR-14, SHAPES project. The funders had no role in study design, data collection and analysis, decision to publish, or preparation of the manuscript.

## Data Availability

All alignments generated in this study are available from the authors upon request.

## 

**Conflict of interest:** None declared.

## Supplementary Material

vez032_Supplementary_Data
