# Supplementary material for: Modular nature of simian foamy virus genomes and their evolutionary history
Source: Virus Evol. 2019 Oct 16;5(2):vez032. doi: 10.1093/ve/vez032 (PMC6795992; doi:10.1093/ve/vez032)
Supplement: vez032_Supplementary_Data [file vez032_supplementary_data.docx]

**Supplemental Table 1 – list of primers used in this study to amplify the *env* gene.**

Consensus or degenerate primers were designed on an alignment of several simian foamy viruses. The site of hybridization is here defined on prototype foamy virus (Genbank: Y07725).

| **PCR** | **Use** | **Direction** | **Sequence (5' -> 3')** | **Site of hybridization (bp)** |
| --- | --- | --- | --- | --- |
| Fragment 1 | External PCR | F | CTGGTCTCCTTCTGTTGGCC | 6957 – 6976 |
|  |  | R | GATATTGTCTTTGGTCYCTAGG | 7790 – 7811 |
|  | Internal PCR | F | GCCAACTTGTCCAGGAGAGG | 6974 – 6993 |
|  |  | R | CCAATGGGATATCRAAGTCAATC | 7762 – 7784 |
| Fragment 2 | External PCR | F | CCWGTCATWGAYTGGAATGT | 7451 – 7470 |
|  |  | R | TTGCYAGCATTCCTTCCATA | 9033 – 9052 |
|  | Internal PCR | F | CCATAATTCTCAAGGAGAGG | 7611 – 7630 |
|  |  | R | GYTGGAGGAAATTTRGGATC | 8780 – 8799 |
| Fragment 3 | External PCR | F | ATGGAATGTATGRATGCTGC | 8651 – 8670 |
|  |  | R | AACATATGCTGGTATWGAGC | 9600 – 9619 |
|  | Internal PCR | F | TAGCAAATATGGAATWGATAG | 8671 – 8691 |
|  |  | R | GTGTCACAAATCACATAATC | 9440 – 9459 |
| Fragment 4 | External PCR | F | ACTCAGCTGGTCAYTTGACC | 9333 – 9352 |
|  |  | R | TGGAACTTGAGGTTCATAGC | 9702 – 9721 |
|  | Internal PCR | F | CTTAGTAAAGGTYCAACATCCT | 9352 – 9373 |
|  |  | R | ACACTGGGTACATAAGCTGG | 9608 – 9627 |
| Fragment 5 | External PCR | F | GTACAAATAGTCTCTCCATGTGG | 9461 – 9483 |
|  |  | R | ACAGCCATTTCGGRGGWTCC | 10872 – 10891 |
|  | Internal PCR | F | GCTCCATATATTCAAGTTTCAGC | 9533 – 9555 |
|  |  | R | CATGGSGTCACCAYTGGAAG | 10769 – 10788 |

**Supplemental Table 2. The numbers of average total per-lineage substitutions per site and their corresponding evolutionary timescales of *env_cenSu_* and their hosts.**

| **Clade/Divergence event*** | | **Substitutions (Substitutions per site)** | | | **Evolutionary timescale (million years)** | | | **Reference** |
| --- | --- | --- | --- | --- | --- | --- | --- | --- |
|  |  | **Median** | **Upper bound 95% HPD** | **Lower bound 95% HPD** | **Median** | **Upper bound 95% HPD** | **Lower bound 95% HPD** |  |
| **Foamy virus** *(env* gene*)* | | | | | | | | |
| Clade I | Cpz-specific SFVs/SFVggo† | 0.322 | 0.271 | 0.380 | 8.336 | 6.185 | 10.658 | NA |
|  | SFVmmu/SFVmcy† | 0.123 | 0.098 | 0.151 | 1.528 | 1.006 | 2.117 |  |
|  | SFVmsp (North)/SFVmsp (South) | 0.101 | 0.079 | 0.126 | 1.075 | 0.546 | 1.708 |  |
|  | Mac-specific SFV/SFVmsp | 0.307 | 0.254 | 0.371 | 7.638 | 5.129 | 10.451 |  |
|  | OWM SFVs | 0.363 | 0.307 | 0.420 | 10.271 | 7.304 | 13.187 |  |
|  | OWM SFVs/SFVppy | 0.495 | 0.419 | 0.579 | 17.726 | 13.282 | 22.965 |  |
|  | OWMA SFVs | 0.593 | 0.504 | 0.698 | 24.391 | 18.853 | 32.176 |  |
| Clade II | Cpz-specific SFVs/SFVggo† | 0.335 | 0.278 | 0.395 | 8.900 | 6.754 | 11.526 |  |
|  | SFVmmu/SFVmcy† | 0.144 | 0.114 | 0.177 | 2.006 | 1.295 | 2.799 |  |
|  | SFVmsp (North)/SFVmsp (South) | 0.059 | 0.043 | 0.075 | 0.415 | 0.171 | 0.718 |  |
|  | SFVmsp/SFVcae, cni | 0.262 | 0.222 | 0.307 | 5.792 | 4.090 | 7.753 |  |
|  | OWM SFVs | 0.318 | 0.272 | 0.370 | 8.153 | 5.940 | 10.450 |  |
|  | OWMA SFVs | 0.612 | 0.507 | 0.738 | 25.697 | 18.674 | 34.773 |  |
| Clade I and II | | 0.666 | 0.583 | 0.755 | 29.892 | 24.230 | 36.743 |  |
| OWMA/NWM SFVs | | 0.837 | 0.717 | 0.969 | 44.887 | 34.303 | 56.167 |  |
| SFVs/fereungulata FVs (Root)† | | 1.268 | 1.070 | 1.488 | 93.369 | 77.382 | 110.881 |  |
| **Host** | | | | | | | | |
| Ptr/Psc | | NA | | | 0.38 | 0.30 | 0.48 | Bjork et al, 2011 |
| Cpz | |  |  |  | 1.03 | 0.81 | 1.26 |  |
| Cpz/Ggo† | |  |  |  | 8.30 | 6.58 | 10.07 | Perelman et al., 2011 |
| Cpz/Ggo/Ppy | |  |  |  | 16.52 | 13.45 | 19.68 |  |
| Mmu/Mcy† | |  |  |  | 1.80 | 1.09 | 2.55 |  |
| Msp (North)/Msp (South) | |  |  |  | 0.80 | 0.56 | 1.40 | Telfer et al. 2003 |
| Mac/Msp | |  |  |  | 8.13 | 6.69 | 9.68 | Perelman et al., 2011 |
| OWMs | |  |  |  | 11.50 | 9.18 | 13.85 |  |
| OWMAs | |  |  |  | 31.56 | 25.66 | 37.88 |  |
| OWMAs/NWMs | |  |  |  | 43.47 | 38.55 | 48.36 |  |
| Simians/fereungulates† | |  |  |  | 98.9 | 96.2 | 101.6 | Bininda-Emonds et al., 2007 |

*SFV: simian foamy virus; Cpz, Chimpanzee (including Psc, *Pan troglodytes schweinfurthii*; Pve, *Pan troglodytes verus*; and Ptr, *Pan troglodytes troglodytes*); Ggo, *Gorilla gorilla gorilla* gorilla; Ppy, *Pongo pygmaeus* orangutan; Mmu, *Macaca mulatta* macaque; Mcy, *Macaca cyclopis* macaque; Mac, macaque; Msp, *Mandrillus sphinx* mandrill; cae, *Chlorocebus aethiops* grivet; cni, *Cercopithecus nictitans* greater spot-nosed monkey; OWM, Old World monkey (including macaque, mandrill, grivet, and greater spot-nosed monkey); OWMA, Old World monkey and ape; NWM, New World Monkey.

†Divergent events used to estimate the time-dependent rate phenomenon model. The timescales of the divergent events were inferred from those of their hosts, and could be found in the ‘Host’ subsection of the table down below.

‘NA’ = Not applicable

**Supplementary Table 3. Codon frequency table**

| **Codon** | **Genomic region** | | |
| --- | --- | --- | --- |
|  | ***env_LP/5’SU_* + *env_3’SU/TM_*** | ***env_cenSU_* variant I** | ***env_cenSU_* variant II** |
| UUU(F) | 1.68 | 1.57 | 1.54 |
| UUC(F) | 0.32 | 0.43 | 0.46 |
| UUA(L) | 1.75 | 3.29 | 3.34 |
| UUG(L) | 1.2 | 0.88 | 0.77 |
| CUU(L) | 0.92 | 0.75 | 1.01 |
| CUC(L) | 0.36 | 0.25 | 0.22 |
| CUA(L) | 0.99 | 0.74 | 0.5 |
| CUG(L) | 0.79 | 0.09 | 0.15 |
| AUU(I) | 1.32 | 1.41 | 1.17 |
| AUC(I) | 0.42 | 0.33 | 0.23 |
| AUA(I) | 1.26 | 1.26 | 1.6 |
| AUG(M) | 1 | 1 | 1 |
| GUU(V) | 1.31 | 1.18 | 1.63 |
| GUC(V) | 0.47 | 0.74 | 0.25 |
| GUA(V) | 1.31 | 1.92 | 1.53 |
| GUG(V) | 0.91 | 0.15 | 0.6 |
| UCU(S) | 1.76 | 1.62 | 1.86 |
| UCC(S) | 0.58 | 0.86 | 0.91 |
| UCA(S) | 1.34 | 0.97 | 1.19 |
| UCG(S) | 0.1 | 0.08 | 0 |
| CCU(P) | 1.99 | 1.86 | 2.12 |
| CCC(P) | 0.43 | 0.48 | 0.68 |
| CCA(P) | 1.54 | 1.54 | 1.17 |
| CCG(P) | 0.03 | 0.12 | 0.03 |
| ACU(T) | 2.16 | 2.08 | 2.23 |
| ACC(T) | 0.44 | 0.68 | 0.48 |
| ACA(T) | 1.27 | 1.2 | 1.03 |
| ACG(T) | 0.14 | 0.05 | 0.26 |
| GCU(A) | 1.99 | 2.01 | 2.22 |
| GCC(A) | 0.71 | 1.31 | 0.44 |
| GCA(A) | 1.17 | 0.68 | 1.31 |
| GCG(A) | 0.13 | 0 | 0.04 |
| UAU(Y) | 1.68 | 1.79 | 1.78 |
| UAC(Y) | 0.32 | 0.21 | 0.22 |
| CAU(H) | 1.72 | 1.65 | 1.54 |
| CAC(H) | 0.28 | 0.35 | 0.46 |
| CAA(Q) | 1.42 | 1.48 | 1.47 |
| CAG(Q) | 0.58 | 0.52 | 0.53 |
| AAU(N) | 1.65 | 1.85 | 1.75 |
| AAC(N) | 0.35 | 0.15 | 0.25 |
| AAA(K) | 1.17 | 1.32 | 1.47 |
| AAG(K) | 0.83 | 0.68 | 0.53 |
| GAU(D) | 1.32 | 1.78 | 1.69 |
| GAC(D) | 0.68 | 0.22 | 0.31 |
| GAA(E) | 1.51 | 1.75 | 1.51 |
| GAG(E) | 0.49 | 0.25 | 0.49 |
| UGU(C) | 1.55 | 1.71 | 1.37 |
| UGC(C) | 0.45 | 0.29 | 0.63 |
| UGG(W) | 1 | 1 | 1 |
| CGU(R) | 0.22 | 0.02 | 0.19 |
| CGC(R) | 0.03 | 0 | 0.02 |
| CGA(R) | 0.5 | 0.36 | 0.28 |
| CGG(R) | 0.19 | 0.12 | 0.42 |
| AGU(S) | 1.55 | 1.95 | 1.66 |
| AGC(S) | 0.67 | 0.51 | 0.38 |
| AGA(R) | 3.31 | 3.45 | 3.42 |
| AGG(R) | 1.76 | 2.04 | 1.67 |
| GGU(G) | 0.76 | 0.73 | 0.71 |
| GGC(G) | 0.42 | 0.06 | 0.14 |
| GGA(G) | 2.11 | 2.61 | 2.34 |
| GGG(G) | 0.71 | 0.59 | 0.81 |
